# Construction and verification of a depression risk prediction model for cancer survivors based on NHANES 2005–2018

**DOI:** 10.1097/MD.0000000000046098

**Published:** 2025-11-28

**Authors:** Huijuan Zhang, Ansi Qi, Hao Zhang, Wenhong Cheng, Lanlan Wang

**Affiliations:** a Department of Psychological Medicine, Shanghai General Hospital, Shanghai Jiao Tong University School of Medicine, Shanghai, China; b Shanghai Mental Health Center, Shanghai Jiao Tong University School of Medicine, Shanghai, China; c Shanghai Key Laboratory of Emotions and Affective Disorders, Shanghai Jiao Tong University School of Medicine, Shanghai, China.

**Keywords:** cancer survivors, depression, nomogram, predictive model

## Abstract

Depression is a significant issue among cancer survivors, and timely identification of depressive symptoms is crucial. This study aims to develop and evaluate a predictive model for depression risk in cancer survivors. 2279 cancer survivors in the National Health and Nutrition Examination Survey were included. Participants were randomly allocated to training and validation sets in a 7:3 ratio. Least absolute shrinkage and selection operator and multivariate logistic regression identified independent predictors of depression (defined as PHQ-9 ≥ 10), which were used to develop a nomogram. Model performance was assessed using receiver operator characteristic, the calibration curve, the Hosmer–Lemeshow test, and the decision curve analysis. Seven variables were identified as significant predictors for depression in cancer survivors: age, education level, poverty-to-income ratio, smoking status, congestive heart failure, sleep disorders, and number of cancers. A nomogram was developed using the 7 predictors. The area under the curve for the model’s training and validation sets was 0.802 (95% confidence interval [CI]: 0.767–0.836) and 0.794 (95% CI: 0.740–0.849), respectively. Internal validation via bootstrapping yielded an optimism-corrected area under the curve of 0.812 (95% CI: 0.784–0.840). Calibration curves and the Hosmer–Lemeshow test illustrated the model has favorable calibration capability. Decision curve analysis results demonstrated that the model has satisfactory clinical application. This study developed a nomogram to predict depression risk in cancer survivors, demonstrating potential clinical utility for identifying high-risk individuals.

## 1. Introduction

Owing to advancements in early detection and therapeutic strategies for cancer, there has been a notable rise in the number of cancer survivors in recent years.^[1]^ Despite the potential for longer life expectancy, many cancer survivors still struggle with numerous negative influences caused by cancer. These include persistent physical discomfort, treatment-related side effects, and the impact of the tumor on their work, social relationships, and lifestyle.^[2]^ Consequently, depression is becoming a prominent issue among cancer survivors.^[3]^ In comparison with the general population, individuals with cancer have a prevalence of major depression that is approximately twice as high.^[4]^ A meta-analysis indicated that, in non-palliative settings, the prevalence of depression in cancer patients was estimated to be 16.3% (95% confidence interval [CI]: 13.4–19.5%).^[5]^ Moreover, depression is prevalent and occurs throughout the survivorship trajectory.^[6]^

Depression comorbid with cancer has various adverse impacts on patients’ health and well-being. Depressive symptoms impair patients’ quality of life and reduce their adherence to treatment and subsequent follow-up care, potentially leading to accelerated disease progression.^[7]^ Besides, depression is found to be linked with increased inflammatory responses and impaired immune function.^[8]^ Furthermore, there is an established association between depression and a higher incidence of suicidal ideation.^[9]^ In the long term, depression has also been identified as an important risk factor for both cancer-specific mortality and all-cause mortality.^[10]^ Therefore, it is imperative to recognize depression in cancer survivors promptly.

However, depressive symptoms among cancer survivors are frequently under-recognized.^[11]^ Symptoms are often misinterpreted as a normal response to cancer diagnosis, or obscured by somatic symptoms such as fatigue. Meanwhile, the factors contributing to depression in cancer survivors are multiple and complex. They include sociodemographic factors such as age, gender, race, educational attainment, and marital status.^[12]^ Lifestyle behaviors, comorbid somatic illnesses, and cancer-related factors such as cancer type have also been reported to be related to depression among cancer patients.^[13]^ A tool that can help to predict the risk of depression among cancer survivors is necessary and useful in clinical practice.

A nomogram serves as a graphical tool designed to visually estimate the likelihood of a specific outcome or to calculate complex statistical relationships. It can simplify complex statistical models into a visual format that allows healthcare providers to make more informed decisions.^[14]^ While machine learning approaches offer potential advantages in predictive accuracy, their complexity leads to black-box models, limiting interpretability and hindering clinical adoption. In contrast, nomograms provide a transparent and intuitive tool that transforms statistical predictions into actionable visual tools. Previous studies established some nomograms to predict depression risk in patients with colorectal cancer,^[15]^ breast cancer,^[16,17]^ and lung cancer.^[18]^ However, the models in most studies focused on patients with one specific type of cancer, therefore may lack common predictive validity across cancer types. In addition, previous studies were mainly conducted on inpatients undergoing treatment in the hospital, which may not be representative of cancer survivors in the community. To the best of our knowledge, there is still limited study that has established a predictive tool for depression risk in cancer survivors across various cancer entities. Recently, Zuo and Yang^[19]^ reported a dynamic nomogram based on 4 variables using the National Health and Nutrition Examination Survey (NHANES) database. However, their model did not incorporate several clinically significant variables related to various comorbidities and cancer-related factors such as number of tumors.

Based on previous findings, we hypothesized that a limited set of readily available sociodemographic, clinical, and lifestyle factors could be integrated into a predictive model to effectively identify cancer survivors at high risk of depression. To address this need, this investigation utilized data sourced from NHANES, a large-scale, nationally representative cross-sectional survey conducted in the United States. The objective of the research was to investigate a more comprehensive set of contributing factors and to develop a nomogram for predicting depression risk in adult cancer survivors, in order to assist in the identification and management of individuals at high risk of depression.

## 2. Methods

### 2.1. Study design and data source

This research is a secondary analysis of cross-sectional data from the NHANES. The NHANES is a large-scale national health survey in the United States, performed every 2 years. It gathers extensive health information from a representative sample of the US population. NHANES 2005–2018 was selected as the original dataset for further analysis, which contained seven 2-year survey cycles. NHANES is an open-source database, the survey was approved by the Ethics Review Committee of the National Center for Health Statistics (NCHS), and all participants provided written informed consents. The research was carried out in accordance with the principles outlined in the Declaration of Helsinki.

### 2.2. Participants

The diagnosis of cancer was obtained from self-reported results in the “Medical Conditions Questionnaire” in the NHANES survey. Participants were queried with the question, “Have you ever been told by a doctor or other health professional that you had cancer or a malignancy of any kind?.” Those who answered “yes” were categorized as cancer survivors. The inclusive criteria for the study were participants aged 20 years or older; participants had been diagnosed with cancer or malignancy. The exclusion criteria included individuals under the age of 20 years old; those without a diagnosis of cancer or malignancy; and those who had any missing data on PHQ-9 or other covariates required for analysis. A total of 70,190 survey samples were included for data screening, and among them, 3782 adult patients self-reported a cancer diagnosis. After eliminating subjects with missing or invalid data following the exclusion criteria, A total of 2279 participants were ultimately included for further analysis.

### 2.3. Assessment of depression

Depressive symptoms were measured using the Patient Health Questionnaire (PHQ-9), which is a widely used 9-item questionnaire for assessing depression severity. It includes 9 items such as loss of interest, depressed mood, sleep problems, fatigue, changes in appetite, negative self-image, difficulty concentrating, psychomotor issues, and suicidal ideation. The PHQ-9 scoring system assigns values of 0 to 3 for each item based on symptom frequency, ranging from “not at all” to “nearly every day.” Total scores are calculated by summing all items, with higher scores indicating greater depression severity. In this study, a score of 10 or higher on the PHQ-9 is considered indicative of depression.^[20]^

### 2.4. Data selection and interpretation of predictors

The study incorporated a range of sociodemographic variables associated with depression and cancer, including age, gender, race, educational attainment, and marital status. Age was categorized as 20–44, 45–64, and ≥ 65 years old, consistent with the standard life-course groupings used by the NCHS in the United States.^[21]^ Poverty-to-income ratio (PIR) was calculated as an index of income related to household needs, and was categorized into 3 groups (<1.3, 1.3–3.5, and > 3.5), with lower scores corresponding to poorer economic status.^[22]^ The lifestyle factors encompassed smoking status, drinking status, and sleep duration. Body mass index was also calculated for further analysis. Comorbid chronic diseases were identified through the “Medical Conditions Questionnaire.” Participants who responded “yes” were classified as having the specific diseases listed in the questions.

Cancer-related factors were gathered via the self-reported Medical Conditions Questionnaire. The number of cancers the patient had (1, 2, or multiple) and cancer type were documented. The first cancer type reported was considered as primary cancer and was used for classification based on anatomical site or system^[23]^: skin (non-melanoma), genitourinary, breast, gynecological, digestive/gastrointestinal, skin (unknown kind), and melanoma. The remaining cancer groups, which had fewer respondents, were categorized as group “other.” Age at diagnosis was recorded, and cancer duration was calculated as the time interval between cancer diagnosis and assessment date. In addition, we extracted some significant laboratory data frequently reviewed in cancer survivors, including blood counts, indicators of liver and kidney function, and blood lipids in standard biochemistry profiles.

### 2.5. Statistical analysis

Before the formal analysis, we adjusted the sample statistics using weight variables. We used “SDMVSTRA” and “SDMVPSU” as sampling stratum and primary sampling units, and applied the “WTMEC2YR” survey weight to align the sample with the demographic structure of the American population. For continuous variables, weighted means ± standard deviations (SD) were reported, and comparisons between groups were made using independent samples *t*-tests. Categorical variables were described using frequencies or percentages, and differences between groups were assessed using Pearson chi-square test.

To construct and verify the nomogram model, participants were randomly allocated to the training set or the validation set in a 7:3 proportion. The least absolute shrinkage and selection operator (LASSO) regression analysis and weighted multivariate logistic regression were conducted to confirm the risk factors. Consequently, a nomogram model for predicting depression risk in cancer survivors was developed using the predictors identified. Sensitivity analysis was performed by multiple imputation (m = 20) using the mice package in R. LASSO variable selection and multivariable logistic regression were applied to each imputed dataset, and results were pooled with Rubin’s rules. Results were compared with those from the primary analysis set to confirm robustness. The discriminative performance of both training and validation datasets was evaluated through the calculation of the area under the curve (AUC) in the receiver operating characteristic curve (ROC). Internal validation was performed using bootstrapping with 1000 repetitions to obtain optimism-adjusted performance estimates, providing a more robust evaluation of model performance. The calibration curve was used to assess the calibration performance. The ideal line on the graph is a 45-degree diagonal line, representing the scenario where actual and predicted probabilities are equal. If the calibration curve closely follows this ideal line, it indicates that the nomogram has good calibration and accurate predictive capability. In addition, the Hosmer–Lemeshow goodness-of-fit test was applied to provide further confirmation of the model’s predictive capability. Besides, Decision Curve Analysis (DCA) as conducted to assess the clinical utility of the predictive tool. A *P*-value of <.05 was considered statistically significant. Data analysis was performed using R (Version 4.2.1, R Foundation for Statistical Computing, Vienna, Austria).

## 3. Results

### 3.1. Baseline characteristics

A total of 2279 cancer survivors who met the criteria were included, comprising 241 subjects (10.6%) in the depression group and 2038 subjects (89.4%) in the non-depression group, with a weighted prevalence of 9.0%. Compared with the non-depression group, cancer survivors comorbid with depression were predominantly middle-aged, female, less educated, and had a lower PIR. Besides, these individuals were more likely to have an increased incidence of coronary heart disease, stroke, asthma, arthritis, congestive heart failure, angina, and chronic bronchitis. Subjects in the depression group also exhibited shorter sleep duration and higher risks of sleep disorder and smoking habits. There were significant differences between the 2 groups regarding race, age at cancer diagnosis, cancer type, high-density lipoprotein, blood urea nitrogen, and triglycerides. Detailed information on the weighted data can be found in Table 1. Meanwhile, these 2279 subjects were randomly divided into 2 sets: a training set (n = 1594) and a validation set (n = 685), with a 7:3 ratio. Statistical comparisons between the datasets revealed nonsignificant differences (*P* > .05), confirming the comparability of baseline characteristics between the groups. The profiles of both the training and validation sets are detailed in Table S1, Supplemental Digital Content, https://links.lww.com/MD/Q745.

**Table 1:** Characteristics of cancer survivors with and without depression included in the study.

Variables	Overall N = 2279 (100.0%)*	Depression N = 241 (9.0%)*	Non-depression N = 2038 (91.0%)*	*P*-value†
Age				**<.001**
45–64	707 (38.2%)	107 (53.1%)	600 (36.8%)	
20–44	229 (12.3%)	45 (20.0%)	184 (11.5%)	
≥65	1343 (49.5%)	89 (27.0%)	1254 (51.7%)	
Gender				**.031**
Male	1069 (43.1%)	80 (33.6%)	989 (44.1%)	
Female	1210 (56.9%)	161 (66.4%)	1049 (55.9%)	
Race				**.001**
Non-Hispanic White	1634 (88.0%)	152 (80.0%)	1482 (88.8%)	
Non-Hispanic Black	299 (4.6%)	29 (6.1%)	270 (4.5%)	
Mexican American	132 (2.0%)	32 (4.8%)	100 (1.7%)	
Other Hispanic	131 (2.1%)	14 (2.1%)	117 (2.2%)	
Other - Including Multi-racial	83 (3.3%)	14 (7.0%)	69 (2.9%)	
Education				**<.001**
<9th grade	199 (4.3%)	43 (9.3%)	156 (3.8%)	
9–11th grade	258 (7.7%)	39 (12.1%)	219 (7.3%)	
High school grad or equivalent	513 (20.8%)	55 (26.4%)	458 (20.3%)	
Some college or AA degree	685 (32.5%)	74 (37.7%)	611 (32.0%)	
College graduate or above	624 (34.7%)	30 (14.5%)	594 (36.7%)	
PIR				**<.001**
<1.3	533 (14.6%)	122 (37.6%)	411 (12.3%)	
1.3–3.5	927 (36.6%)	86 (40.4%)	841 (36.2%)	
≥3.5	819 (48.9%)	33 (22.1%)	786 (51.5%)	
Marriage				**<.001**
Married	1323 (62.6%)	98 (48.8%)	1225 (64.0%)	
Widowed	378 (13.3%)	41 (13.1%)	337 (13.4%)	
Divorced	297 (12.1%)	56 (21.3%)	241 (11.2%)	
Separated	71 (2.6%)	16 (6.5%)	55 (2.2%)	
Never married	135 (5.7%)	23 (7.2%)	112 (5.6%)	
Living with partner	75 (3.6%)	7 (3.2%)	68 (3.7%)	
BMI				.10
Underweight (<18.5)	36 (1.7%)	3 (0.9%)	33 (1.7%)	
Normal Weight (18.5–24.9)	595 (27.8%)	61 (24.3%)	534 (28.2%)	
Overweight (25–29.9)	803 (34.9%)	68 (30.2%)	735 (35.3%)	
Obesity (≥30)	845 (35.6%)	109 (44.6%)	736 (34.8%)	
Diabetes				.4
Yes	418 (14.6%)	60 (16.5%)	358 (14.5%)	
No	1783 (81.7%)	171 (78.4%)	1612 (82.1%)	
Borderline	78 (3.6%)	10 (5.2%)	68 (3.5%)	
Coronary heart disease				**.009**
Yes	200 (7.1%)	32 (11.9%)	168 (6.6%)	
No	2079 (92.9%)	209 (88.1%)	1870 (93.4%)	
Stroke				**<.001**
Yes	193 (5.9%)	36 (11.8%)	157 (5.3%)	
No	2086 (94.1%)	205 (88.2%)	1881 (94.7%)	
Asthma				**.029**
Yes	372 (17.2%)	61 (24.9%)	311 (16.4%)	
No	1907 (82.8%)	180 (75.1%)	1727 (83.6%)	
Arthritis				**.004**
Yes	1177 (49.3%)	156 (62.0%)	1021 (48.0%)	
No	1102 (50.7%)	85 (38.0%)	1017 (52.0%)	
Congestive heart failure				**<.001**
Yes	153 (5.5%)	32 (11.1%)	121 (4.9%)	
No	2126 (94.5%)	209 (88.9%)	1917 (95.1%)	
Angina				**.003**
Yes	124 (4.3%)	22 (9.0%)	102 (3.8%)	
No	2155 (95.7%)	219 (91.0%)	1936 (96.2%)	
Heart attack				.073
Yes	205 (6.8%)	31 (9.6%)	174 (6.5%)	
No	2074 (93.2%)	210 (90.4%)	1864 (93.5%)	
Chronic bronchitis				**.001**
Yes	213 (10.0%)	44 (17.7%)	169 (9.2%)	
No	2066 (90.0%)	197 (82.3%)	1869 (90.8%)	
Thyroid problem				.5
Yes	442 (19.9%)	52 (22.0%)	390 (19.7%)	
No	1837 (80.1%)	189 (78.0%)	1648 (80.3%)	
Sleep duration				**.001**
≤7 h	1288 (54.4%)	159 (67.2%)	1129 (53.2%)	
7–9 h	873 (41.2%)	67 (27.6%)	806 (42.6%)	
>9 h	118 (4.3%)	15 (5.2%)	103 (4.2%)	
Sleep disorder				**<.001**
Yes	816 (38.5%)	155 (70.7%)	661 (35.3%)	
No	1463 (61.5%)	86 (29.3%)	1377 (64.7%)	
Smoking status				**<.001**
Current	355 (15.9%)	85 (38.0%)	270 (13.8%)	
Former	902 (38.4%)	79 (32.1%)	823 (39.0%)	
Never	1022 (45.7%)	77 (29.9%)	945 (47.3%)	
Drinking status				.8
Current	1628 (76.3%)	168 (76.8%)	1460 (76.3%)	
Former	366 (13.5%)	43 (14.4%)	323 (13.4%)	
Never	285 (10.1%)	30 (8.9%)	255 (10.3%)	
Hypertension				.5
Yes	1295 (50.9%)	142 (53.4%)	1153 (50.6%)	
No	984 (49.1%)	99 (46.6%)	885 (49.4%)	
Age at cancer diagnosis	50.49 (16.96)	44.10 (16.49)	51.12 (16.87)	**<.001**
Cancer duration	11.71 (11.40)	11.92 (10.77)	11.69 (11.46)	.8
Number of cancer				.2
1	2048 (89.4%)	212 (87.0%)	1836 (89.6%)	
2	209 (9.5%)	23 (10.6%)	186 (9.4%)	
Multiple	22 (1.1%)	6 (2.5%)	16 (1.0%)	
Cancer type				**<.001**
Skin (non-melanoma)	387 (22.8%)	27 (16.9%)	360 (23.4%)	
Genitourinary	381 (11.2%)	24 (8.4%)	357 (11.5%)	
Breast	351 (14.0%)	38 (13.9%)	313 (14.0%)	
Gynecological	309 (13.4%)	65 (27.4%)	244 (12.1%)	
Digestive/Gastrointestinal	230 (7.8%)	32 (8.6%)	198 (7.7%)	
Skin (unknown kind)	176 (9.1%)	17 (7.0%)	159 (9.3%)	
Melanoma	141 (7.6%)	11 (5.8%)	130 (7.8%)	
Other	304 (14.1%)	27 (12.0%)	277 (14.4%)	
Total cholesterol	5.09 (1.15)	5.19 (1.23)	5.08 (1.15)	.3
High-density lipoprotein (HDL)	1.42 (0.46)	1.31 (0.43)	1.44 (0.46)	**<.001**
Hemoglobin A1c	5.76 (0.84)	5.88 (1.14)	5.75 (0.81)	.13
Albumin	5.50 (2.34)	4.90 (2.40)	5.56 (2.32)	**.001**
Creatine	42.27 (3.21)	41.97 (3.64)	42.30 (3.17)	.3
Blood urea nitrogen (BUN)	83.53 (38.20)	81.21 (37.82)	83.76 (38.24)	.4
Aspartate aminotransferase (AST)	25.61 (11.86)	26.11 (16.00)	25.56 (11.38)	.6
Alanine aminotransferase (ALT)	23.91 (15.71)	25.80 (22.12)	23.73 (14.92)	.15
Triglycerides	1.84 (1.74)	2.11 (1.53)	1.81 (1.76)	.053

BMI = body mass index, PIR = poverty-to-income ratio

* n (unweighted) (% (weighted)); mean (SD) or frequency (percentage).

† Pearson’s *X*^2^: Rao & Scott adjustment; design-based *t*-test.

### 3.2. Predictors screening and development of nomogram

**Figure 1.**
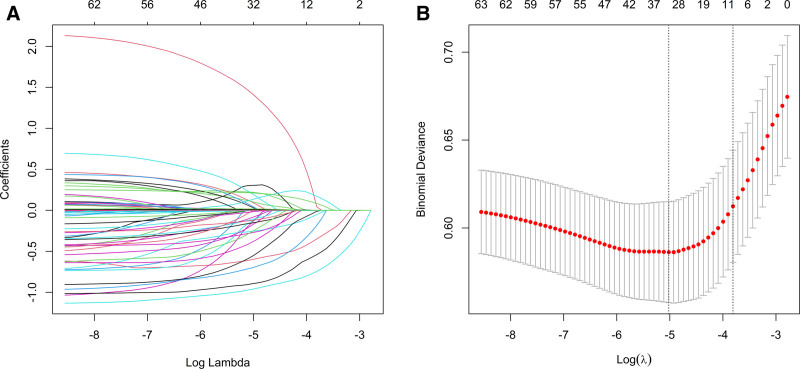
(A) LASSO regression coefficient path diagram. (B) LASSO regression cross-validation curves.

Predictor selection was performed using LASSO regression with 10-fold cross-validation. The optimal penalty parameter (lambda) was determined using the one-standard-error rule (lambda.1se). A total of 62 predictor variables were initially considered for inclusion in the model, encompassing demographic, socioeconomic, comorbidity, cancer-related, and biochemical characteristics. This process resulted in a subset of 11 candidate predictors retaining non-zero coefficients (Fig. 1), with the complete set of LASSO coefficients provided in Table S2, Supplemental Digital Content, https://links.lww.com/MD/Q745. No additional manual exclusion of borderline variables was performed after the automated LASSO selection process. The results of LASSO were further validated by weighted multiple logistic regression analysis, which indicated that seven factors were independently associated with depression (Table 2). Risk factors included middle age, lower educational attainment, lower PIR, congestive heart failure, sleep disorders, current smoking, and multiple cancer sites. The absolute risk differences for the 7 predictors are presented in Table S3, Supplemental Digital Content, https://links.lww.com/MD/Q745, providing a clinically intuitive measure of the difference in depression risk between comparison groups.

**Table 2:** Results of multivariate logistic regression for variables identified by LASSO.

Variables	OR	95% CI	*P*-value
Age			**<.001**
45–64	—	—	
20–44	0.52	0.25, 1.07	
≥65	0.39	0.23, 0.64	
Education			**.013**
<9th grade	—	—	
9–11th grade	0.43	0.18, 1.06	
High School graduate or equivalent	0.62	0.26, 1.50	
Some college or AA degree	0.65	0.28, 1.54	
College graduate or above	0.26	0.10, 0.67	
PIR			**<.001**
<1.3	—	—	
1.3–3.5	0.55	0.32, 0.96	
≥3.5	0.27	0.15, 0.51	
Congestive heart failure			**<.001**
Yes	—	—	
No	0.29	0.15, 0.60	
Sleep disorder			**<.001**
Yes	—	—	
No	0.24	0.15, 0.38	
Smoking status			**.012**
Current	—	—	
Former	0.42	0.20, 0.85	
Never	0.41	0.22, 0.77	
Number of cancer			**.035**
1	—	—	
2	1.65	0.75, 3.61	
Multiple	6.76	1.33, 34.3	
Age at cancer diagnosis	0.99	0.97, 1.01	.3
Cancer type			.5
Skin (non-melanoma)	—	—	
Genitourinary	1.36	0.56, 3.30	
Breast	1.07	0.43, 2.63	
Gynecological	1.19	0.49, 2.89	
Digestive/Gastrointestinal	0.81	0.38, 1.71	
Skin (unknown kind)	0.97	0.38, 2.50	
Melanoma	0.77	0.22, 2.66	
Other	0.64	0.24, 1.71	
Hemoglobin A1c	1.16	0.92, 1.47	.2
Aspartate aminotransferase (AST)	1.01	1.00, 1.02	.10

CI = confidence interval, LASSO = least absolute shrinkage and selection operator, OR = odds ratio, PIR = poverty-to-income ratio.

**Figure 2.**
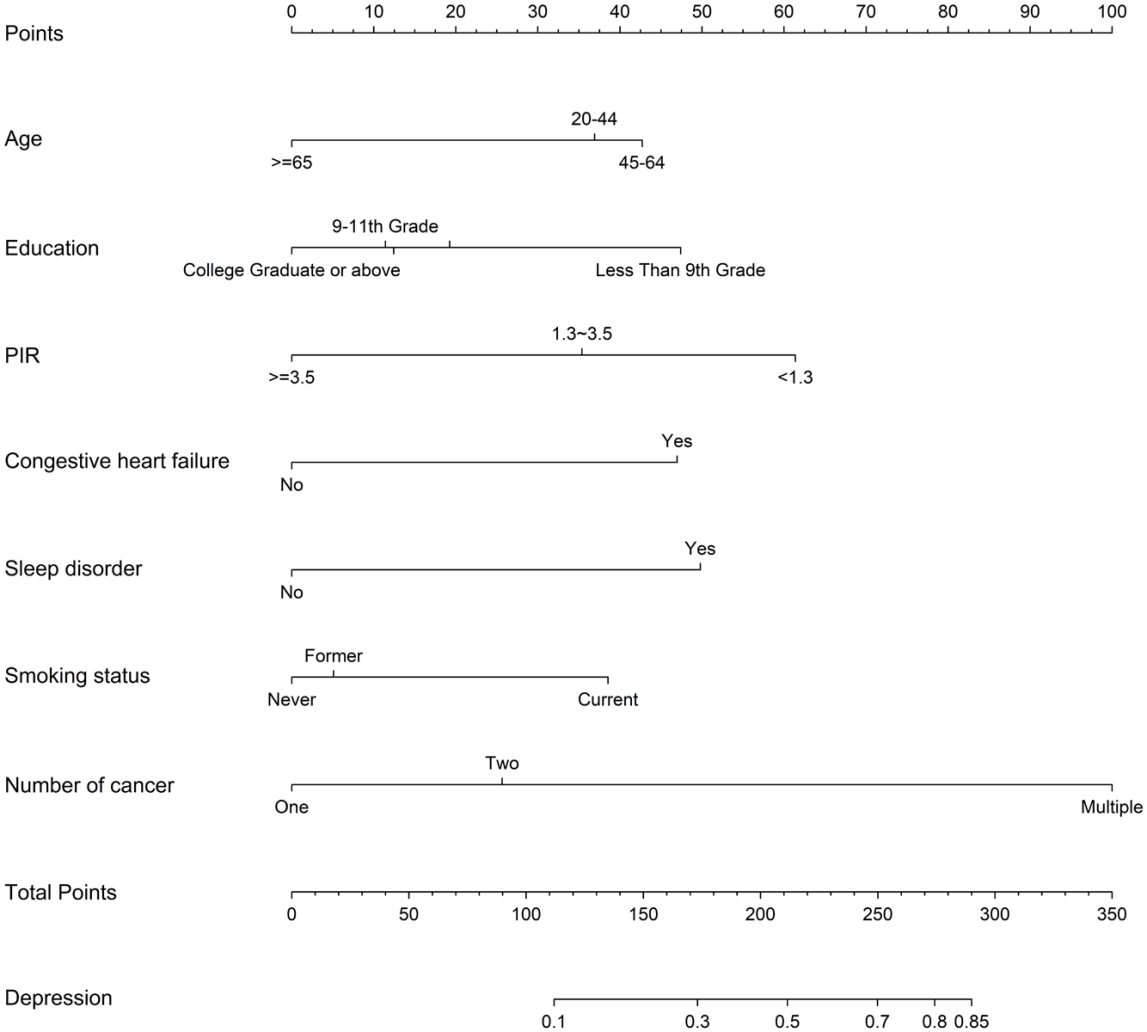
Nomogram for predicting depression risk in cancer survivors.

Results from the multiple imputation sensitivity analysis strongly support the robustness of our primary complete-case findings. All 7 original predictors retained statistical significance, with negligible changes in the effect sizes and confidence intervals. No additional predictors were identified in the sensitivity analysis (Table S4, Supplemental Digital Content, https://links.lww.com/MD/Q745). Based on these 7 identified predictors, a predictive nomogram was constructed to estimate depression risk among cancer survivors, as demonstrated in Figure 2. Each predictor corresponds to a specific value on the upper point scale. These values are summed and the total is aligned with the total points axis, which corresponds to a predicted probability of depression on the final risk axis. For instance, a 70-year-old patient with a primary school education, a PIR of 1.0, no congestive heart failure, with sleep disorder, never smoked, and one cancer site, would have a total point value of 202, indicating an approximately 48% risk of depression.

### 3.3. Validation of the predictive model

**Figure 3.**
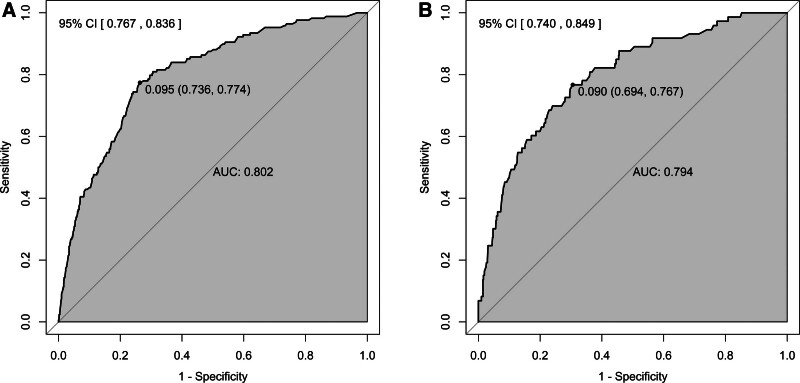
Receiver operating characteristic (ROC) analysis results for the training set (A) and validation set (B).

The predictive performance of the nomogram was assessed through receiver operating characteristic (ROC) analysis, with both training and validation cohorts undergoing independent evaluation. The model demonstrated excellent discrimination, with area under the curve measurements (AUC) of 0.802 (95% CI: 0.767–0.836) in the training set and 0.794 (95% CI: 0.740–0.849) in the validation set, indicating strong predictive performance, as illustrated in Figure 3. To provide a more robust estimate of model performance, bootstrap internal validation with 1000 repetitions was performed, yielding an optimism-corrected AUC of 0.812 (95% CI: 0.784–0.840).

**Figure 4.**
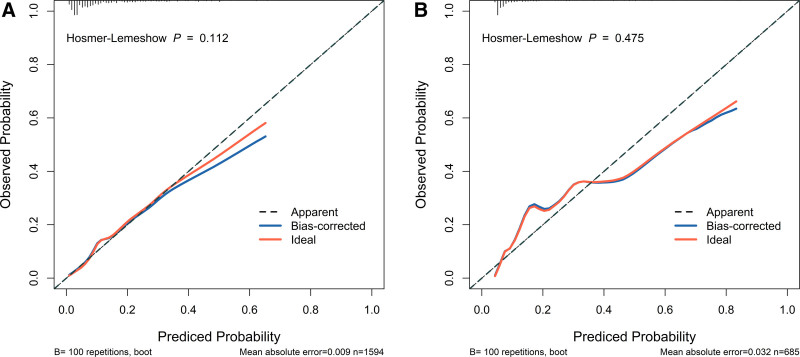
Calibration curves demonstrating the predictive accuracy of the nomogram model in the training set (A) and validation set (B).

The Calibration Curves were utilized to assess prediction accuracy in both sets. Figure 4 illustrates the strong agreement between predicted probabilities and observed outcomes, with calibration curves closely following the 45-degree reference line in both cohorts. Quantitatively, the calibration slope was 1.00 with an intercept approximating 0 in the training set, while in the validation set the slope was 1.01 with an intercept of −0.03. The Hosmer–Lemeshow test further confirmed model accuracy, showing nonsignificant *P*-values of .112 (training set) and .475 (validation set), indicating satisfactory calibration performance across both datasets.

**Figure 5.**
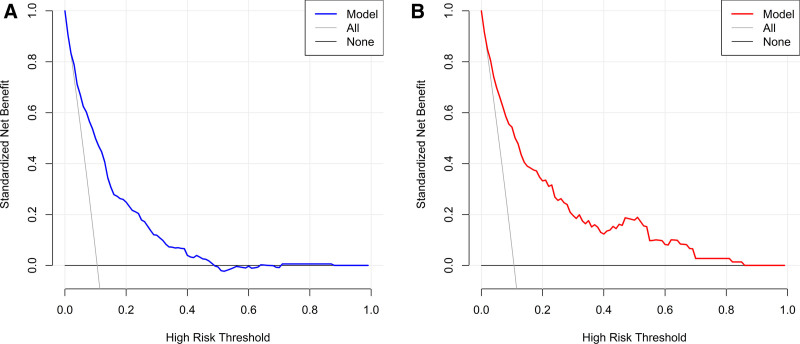
The decision curve analysis (DCA) for the training set (A) and validation set (B).

Decision curve analysis was conducted to assess the clinical utility of the predictive tool, analyzing net benefits across a range of probability thresholds. The graphical representation displays threshold probabilities on the x-axis and corresponding net benefits on the y-axis, with curves illustrating the comparative clinical value of different predictors. As demonstrated in Figure 5, the analysis revealed favorable clinical effectiveness of the proposed model in both datasets, suggesting its practical value in clinical decision-making.

## 4. Discussion

This study employed nationally representative data from the NHANES 2005–2018 to develop and validate a predictive nomogram for depression risk assessment among cancer survivors. Multivariable analysis identified 7 significant predictors: middle-aged status, limited educational attainment, lower poverty-income ratio, current smoking, concurrent congestive heart failure, sleep disturbance comorbidity, and multiple cancer sites. The model demonstrated good discriminative capacity and clinical applicability through comprehensive validation procedures.

This study indicated that middle-aged cancer survivors are more likely to experience depression, which is consistent with prior research findings. Compared to elderly individuals, those diagnosed at working age may encounter a more negative impact of the disease on their occupation and social relationships, resulting in a higher prevalence of depression.^[24,25]^ Meanwhile, our research found that lower income may be a robust predictor of depression among cancer survivors. In line with previous studies, a lack of adequate economic resources can impose significant financial pressures on patients. The financial burden of medical expenses may deteriorate their family’s financial stability, and restrict their access to timely and superior treatment options.^[26]^ Consequently, this can intensify the psychological burden experienced by patients, potentially triggering or exacerbating depressive symptoms.^[27]^ The present study also demonstrated that lower educational attainment may be a significant predictor of depression in cancer survivors. Consistent with the findings of this study, research on prostate cancer patients revealed that individuals with lower educational attainment exhibited a 1.86-fold elevated risk of depression compared to those with higher levels of education.^[28]^ Limited educational attainment can impede their ability to comprehend complex medical information and develop effective coping strategies, which are essential for addressing the emotional challenges posed by cancer.^[29]^

The presence of comorbid chronic conditions might elevate the risk of depression among cancer survivors. A previous review summarized that the risk of depression is consistently higher (up to 1.7 times) in patients comorbid with chronic diseases than in patients without these comorbidities.^[12]^ In this research, we investigated the link between multiple chronic comorbidities and the likelihood of developing depression in individuals with a history of cancer. Notably, we identified congestive heart failure as a significant indicator of depression risk in cancer patients. Previous studies have consistently demonstrated that patients with heart failure are more likely to experience depression, with up to 30% of these individuals experiencing depressive symptoms.^[30]^ Congestive heart failure is one of the most frequently reported comorbidities following a cancer diagnosis,^[31]^ particularly among those who have undergone radiotherapy in conjunction with chemotherapy.^[32]^ However, the exact mechanism of comorbid depression and heart failure in cancer patients remains unclear. We speculate that heart failure may impair physical functioning and diminish overall quality of life. This decline in well-being can lead to a negative impact on daily moods and consequently cause depression.^[33]^ Additionally, shared biological mechanisms, such as neuroendocrine dysregulation,^[34]^ and elevated inflammatory markers (e.g., C-reactive protein and interleukin-6)^[35,36]^, may contribute to this association. Nevertheless, it should be emphasized that these mechanisms remain speculative in cancer survivors. More researches are required to confirm our results and elucidate these pathways and clarify how cardiac function influences mood in this population.

Our study identified a strong link between sleep disorders and depression in cancer survivors. Those without sleep disorders exhibited a significantly lower risk of depression. These results align with previous findings. Evidence from a recent systematic review and meta-analysis highlights the high frequency of sleep quality impairment among individuals with cancer, demonstrating a combined prevalence estimate of 57.4% (95% CI: 53.3–61.6%).^[37]^ Meta-regression analyses have demonstrated a positive correlation between the prevalence of poor sleep quality and comorbid depression.^[37]^ Sleep disturbances can contribute to severe fatigue and cognitive deficits among cancer patients, and disrupt their metabolic systems and neuroendocrine regulation. These factors can in turn impair patients’ quality of life and exacerbate depressive symptoms.^[38,39]^ Besides, aligning with existing literature,^[40]^ our findings found reduced depression risk among never-smokers and former smokers relative to active smokers in the cancer survivors. One possible mechanism is that smoking is associated with elevated inflammation, which can lead to immunological changes in the brain and subsequent depression.^[41]^

In the present study, we also explored some cancer-specific risk factors related to depression. After screening, we found that the number of tumors was a valid predictor of depression in cancer survivors. Individuals with multiple cancer sites exhibit a significantly elevated risk of depression compared to those with a single cancer site (OR = 7.51, 95% CI: 1.66–33.90). A matched-cohort study on Japanese cancer patients has similar findings, depression risk is highest in patients with multiple cancers in comparison to cancer-free individuals.^[42]^ Second primary malignancy and cancer metastasis are very common in cancer survivors, more cancer sites may indicate a worse cancer stage and worse health condition. Several studies have confirmed that advanced cancer stage is linked to elevated depression rates across different cancer types.^[12,13]^ Multiple tumors can lead to higher disease burden, more complex treatments, and greater somatic discomforts, multiple tumors can cause increased inflammation and hormonal imbalances, all of these factors may collectively lead to an elevated occurrence of depression.

A few studies have established some predictive tools for assessing the risk of depression in patients with cancer. Consistent with the contemporaneous work of Zuo and Yang (2025),^[19]^ our study also identified same variables such as lower PIR and sleep disorders as predictors of depression in cancer survivors. Furthermore, our model incorporated a more comprehensive set of clinically relevant predictors, specifically congestive heart failure, smoking status, and multiple cancer sites, which were not included in their final model. These additions enhance the clinical utility and risk stratification capability of our tool. One study predicted the risk of postoperative anxiety and depression in colorectal cancer patients, in line with our findings, lower income and more comorbidity are also predictors of depression in this population.^[15]^ In another study on breast cancer patients, lower income is also one of the predictors of depression risk.^[16]^ Nevertheless, in comparison with cancer survivors, the mental status of inpatients undergoing treatment may be more influenced by treatment-related factors such as pain, postoperative complications, and adjunctive treatment.^[15,43]^ A study has constructed a logistic model of depressive symptoms among cancer patients based on the NHANES database. However, the regression equation comprised over 20 variables and was therefore challenging to apply in clinical practice. Meanwhile, the calibration capability and clinical efficacy of the model have not been validated.^[44]^ In the present study, we employed the LASSO regression to filter the model factors prior to conducting multivariable logistic regression analysis. This approach reduced the risk of overfitting and multicollinearity and simplified the model. The present model incorporated 7 factors, all of which are readily obtainable in clinical settings, thus making the model more practicable. Furthermore, the validity and efficacy of the model were determined by the calibration curve and the DCA analysis, thereby ensuring its stability and reliability.

Early detection of depression in cancer survivors is of great significance, as it can not only promote effective management but also enhance patient prognosis. In economically disadvantaged nations and areas with lower income status, depressive symptoms among cancer patients are more likely to be clinically overlooked, particularly as many individuals in these areas are diagnosed at advanced disease stages, face restricted treatment options, and experience poorer clinical outcomes.^[27]^ Moreover, in primary care settings, where many cancer survivors with stable medical conditions are treated, the paucity of psychiatrists is also a predominant issue, resulting in an inadequacy in the screening and assessment of depression. Therefore, a predictive tool to help identify depression risk would be of great value. This study has identified 7 prominent risk factors that are closely linked to depression in cancer survivors and proposes a tool that utilizes a nomogram to score individuals based on the presence or absence of specific risk factors. This tool can help in recognizing populations vulnerable to depression risk and the delivery of more precise and timely interventions. Greater attention should be paid to populations with one or more of the aforementioned factors.

Inevitably, several limitations should be acknowledged in the present research. First, the data on medical conditions and depressive symptoms in NHANES are derived from patient self-report questionnaires or self-rating scales. The PHQ-9 is a screening instrument rather than a diagnostic measure for depression. The absence of physician review or check with medical records introduces the possibility of reporting or recall bias, which can compromise the data’s accuracy. Additionally, as NHANES surveys only non-institutionalized individuals, it excludes those who are deceased or severely ill, potentially introducing survivorship bias and underestimating depression prevalence and severity among cancer survivors. Second, due to the cross-sectional design of NHANES, no causal inferences can be drawn from our findings. The associations may be influenced by unmeasured confounding variables. Although the NHANES database is extensive and contains numerous variables, some factors potentially linked to depression in cancer patients are not included, such as detailed psychosocial variables (e.g., social support, stress levels) and treatment-related factors. Future studies might explore the impact of a wider range of factors on mood in cancer survivors to improve the effectiveness of the model. Third, a notable proportion of data was missing, which can be partly attributed to the extensive set of covariates incorporated in our study. This may raise the possibility of selection bias. Reassuringly, our multiple imputation-based sensitivity analysis produced results that were highly consistent with those from the primary analysis, thereby strengthening the validity of our main conclusions. Forth, while this study is internally validated, the lack of external data validation remains a noted limitation. The generalizability of our findings to populations outside the United States remains uncertain, particularly in low- and middle-income countries. Differences in healthcare systems, cultural factors, and survivorship care frameworks could limit their applicability. Future studies are necessary to evaluate the model’s consistency and applicability among diverse populations and cultural settings.

## 5. Conclusions

The present study constructed and verified a visual nomogram designed to assess depression risk in cancer survivors. The model integrates 7 readily obtainable predictors and has demonstrated favorable discriminative efficacy, calibration capability, and clinical applicability. This predictive tool may support healthcare providers in detecting cancer survivors with elevated depression risk, thereby facilitating early assessment and prompting intervention in vulnerable populations. Further external validation and prospective studies are warranted to evaluate its generalizability across diverse populations and healthcare settings, as well as to confirm its clinical utility.

## Acknowledgments

The authors gratefully acknowledge the National Health and Nutrition Examination Survey (NHANES) participants and research personnel for their invaluable contributions to data collection and study implementation.

## Author contributions

**Conceptualization:** Huijuan Zhang, Lanlan Wang.

**Data curation:** Huijuan Zhang.

**Formal analysis:** Huijuan Zhang.

**Methodology:** Huijuan Zhang.

**Resources:** Lanlan Wang.

**Software:** Huijuan Zhang.

**Writing – original draft:** Huijuan Zhang.

**Writing – review & editing:** Huijuan Zhang, Ansi Qi, Hao Zhang, Wenhong Cheng, Lanlan Wang.

## Supplementary Material


