# Supplementary material for: Construction and verification of a depression risk prediction model for cancer survivors based on NHANES 2005–2018
Source: Medicine (Baltimore). 2025 Nov 28;104(48):e46098. doi: 10.1097/MD.0000000000046098 (PMC12662453; doi:10.1097/MD.0000000000046098)
Supplement: Supplementary file 1 [file medi-104-e46098-s001.docx]

Table S1- Characteristics of study participants in the training set and the test set

| **Variables** | **Overall**  N = 2279 (100.0%)^1^ | **Training**  N = 1594 (71.1%)^1^ | **Test** N = 685 (28.9%)^1^ | | **P-Value**^2^ |
| --- | --- | --- | --- | --- | --- |
| **Age** |  |  |  | | >0.9 |
| 45-64 | 707 (38.2%) | 485 (38.3%) | 222 (38.2%) | |  |
| 20-44 | 229 (12.3%) | 159 (12.2%) | 70 (12.6%) | |  |
| >=65 | 1,343 (49.5%) | 950 (49.6%) | 393 (49.2%) | |  |
| **Gender** |  |  |  | | 0.7 |
| Male | 1,069 (43.1%) | 746 (42.8%) | 323 (44.0%) | |  |
| Female | 1,210 (56.9%) | 848 (57.2%) | 362 (56.0%) | |  |
| **Race** |  |  |  | | 0.13 |
| Non-Hispanic White | 1,634 (88.0%) | 1,158 (88.6%) | 476 (86.6%) | |  |
| Non-Hispanic Black | 299 (4.6%) | 198 (4.3%) | 101 (5.5%) | |  |
| Mexican American | 132 (2.0%) | 95 (2.1%) | 37 (1.8%) | |  |
| Other Hispanic | 131 (2.1%) | 88 (1.8%) | 43 (3.0%) | |  |
| Other - Including Multi-racial | 83 (3.3%) | 55 (3.3%) | 28 (3.2%) | |  |
| **Education** |  |  |  | | 0.11 |
| Less Than 9th Grade | 199 (4.3%) | 132 (4.1%) | 67 (4.9%) | |  |
| 9-11th Grade | 258 (7.7%) | 181 (7.3%) | 77 (8.8%) | |  |
| High School Graduate or equivalent | 513 (20.8%) | 349 (19.6%) | 164 (23.8%) | |  |
| Some College or AA degree | 685 (32.5%) | 480 (32.9%) | 205 (31.5%) | |  |
| College Graduate or above | 624 (34.7%) | 452 (36.1%) | 172 (31.0%) | |  |
| **PIR** |  |  |  | | 0.2 |
| <1.3 | 533 (14.6%) | 368 (13.7%) | 165 (16.7%) | |  |
| 1.3~3.5 | 927 (36.6%) | 643 (36.3%) | 284 (37.4%) | |  |
| >=3.5 | 819 (48.9%) | 583 (50.0%) | 236 (46.0%) | |  |
| **Marriage** |  |  |  | | 0.7 |
| Married | 1,323 (62.6%) | 923 (62.3%) | 400 (63.4%) | |  |
| Widowed | 378 (13.3%) | 267 (13.6%) | 111 (12.8%) | |  |
| Divorced | 297 (12.1%) | 202 (12.0%) | 95 (12.3%) | |  |
| Separated | 71 (2.6%) | 51 (2.4%) | 20 (2.9%) | |  |
| Never married | 135 (5.7%) | 93 (5.6%) | 42 (6.2%) | |  |
| Living with partner | 75 (3.6%) | 58 (4.1%) | 17 (2.5%) | |  |
| **Body Mass Index** |  |  |  | | 0.084 |
| Underweight (<18.5) | 36 (1.7%) | 24 (1.3%) | 12 (2.7%) | |  |
| Normal Weight（18.5~24.9） | 595 (27.8%) | 425 (28.7%) | 170 (25.8%) | |  |
| Overweight (25~29.9) | 803 (34.9%) | 573 (35.6%) | 230 (33.0%) | |  |
| Obesity (≥30) | 845 (35.6%) | 572 (34.5%) | 273 (38.5%) | |  |
| **Diabetes** |  |  |  | | 0.7 |
| Yes | 418 (14.6%) | 294 (14.5%) | 124 (14.9%) | |  |
| No | 1,783 (81.7%) | 1,246 (82.0%) | 537 (81.0%) | |  |
| Borderline | 78 (3.6%) | 54 (3.4%) | 24 (4.2%) | |  |
| **Coronary heart disease** |  |  |  | | 0.9 |
| Yes | 200 (7.1%) | 148 (7.1%) | 52 (6.9%) | |  |
| No | 2,079 (92.9%) | 1,446 (92.9%) | 633 (93.1%) | |  |
| **Stroke** |  |  |  | | 0.2 |
| Yes | 193 (5.9%) | 145 (6.2%) | 48 (4.9%) | |  |
| No | 2,086 (94.1%) | 1,449 (93.8%) | 637 (95.1%) | |  |
| **Asthma** |  |  |  | | 0.8 |
| Yes | 372 (17.2%) | 267 (17.4%) | 105 (16.8%) | |  |
| No | 1,907 (82.8%) | 1,327 (82.6%) | 580 (83.2%) | |  |
| **Arthritis** |  |  |  | | 0.6 |
| Yes | 1,177 (49.3%) | 844 (49.7%) | 333 (48.2%) | |  |
| No | 1,102 (50.7%) | 750 (50.3%) | 352 (51.8%) | |  |
| **Congestive heart failure** |  |  |  | | 0.11 |
| Yes | 153 (5.5%) | 105 (4.9%) | 48 (7.0%) | |  |
| No | 2,126 (94.5%) | 1,489 (95.1%) | 637 (93.0%) | |  |
| **Angina** |  |  |  | | 0.7 |
| Yes | 124 (4.3%) | 91 (4.1%) | 33 (4.5%) | |  |
| No | 2,155 (95.7%) | 1,503 (95.9%) | 652 (95.5%) | |  |
| **Heart attack** |  |  |  | | >0.9 |
| Yes | 205 (6.8%) | 150 (6.8%) | 55 (6.7%) | |  |
| No | 2,074 (93.2%) | 1,444 (93.2%) | 630 (93.3%) | |  |
| **Chronic bronchitis** |  |  |  | | 0.2 |
| Yes | 213 (10.0%) | 157 (10.6%) | 56 (8.3%) | |  |
| No | 2,066 (90.0%) | 1,437 (89.4%) | 629 (91.7%) | |  |
| **Thyroid problem** |  |  |  | | 0.3 |
| Yes | 442 (19.9%) | 319 (20.6%) | 123 (18.3%) | |  |
| No | 1,837 (80.1%) | 1,275 (79.4%) | 562 (81.7%) | |  |
| **Sleep duration** |  |  |  | | 0.087 |
| <=7h | 1,288 (54.4%) | 920 (55.8%) | 368 (51.1%) | |  |
| 7~9h | 873 (41.2%) | 597 (40.3%) | 276 (43.4%) | |  |
| >9h | 118 (4.3%) | 77 (3.9%) | 41 (5.4%) | |  |
| **Sleep disorder** |  |  |  | | >0.9 |
| Yes | 816 (38.5%) | 569 (38.5%) | 247 (38.7%) | |  |
| No | 1,463 (61.5%) | 1,025 (61.5%) | 438 (61.3%) | |  |
| **Smoking status** |  |  |  | | 0.4 |
| Current | 355 (15.9%) | 245 (15.1%) | 110 (17.9%) | |  |
| Former | 902 (38.4%) | 636 (38.7%) | 266 (37.5%) | |  |
| Never | 1,022 (45.7%) | 713 (46.2%) | 309 (44.6%) | |  |
| **Drinking status** |  |  |  | | 0.2 |
| Current | 1,628 (76.3%) | 1,158 (77.6%) | 470 (73.3%) | |  |
| Former | 366 (13.5%) | 246 (12.8%) | 120 (15.3%) | |  |
| Never | 285 (10.1%) | 190 (9.6%) | 95 (11.5%) | |  |
| **Hypertension** |  |  |  | | 0.9 |
| Yes | 1,295 (50.9%) | 896 (50.8%) | 399 (51.1%) | |  |
| No | 984 (49.1%) | 698 (49.2%) | 286 (48.9%) | |  |
| **Age at cancer diagnosis** | 50.49 (16.96) | 50.46 (16.82) | 50.57 (17.30) | | >0.9 |
| **Cancer duration** | 11.71 (11.40) | 11.87 (11.35) | 11.33 (11.51) | | 0.5 |
| **Number of cancer** |  |  |  | | 0.2 |
| One | 2,048 (89.4%) | 1,423 (88.5%) | 625 (91.5%) | |  |
| Two | 209 (9.5%) | 155 (10.3%) | 54 (7.7%) | |  |
| Multiple | 22 (1.1%) | 16 (1.2%) | 6 (0.9%) | |  |
| **Cancer type** |  |  |  | | 0.3 |
| Skin (non-melanoma) | 387 (22.8%) | 285 (24.2%) | 102 (19.3%) | |  |
| Genitourinary | 381 (11.2%) | 270 (11.3%) | 111 (10.9%) | |  |
| Breast | 351 (14.0%) | 250 (14.1%) | 101 (13.7%) | |  |
| Gynecological | 309 (13.4%) | 205 (12.3%) | 104 (16.4%) | |  |
| Digestive/Gastrointestinal | 230 (7.8%) | 157 (7.8%) | 73 (7.7%) | |  |
| Skin (unknown kind) | 176 (9.1%) | 123 (8.8%) | 53 (10.0%) | |  |
| Melanoma | 141 (7.6%) | 101 (7.7%) | 40 (7.4%) | |  |
| Other | 304 (14.1%) | 203 (13.9%) | 101 (14.7%) | |  |
| **Total cholesterol** | 5.09 (1.15) | 5.11 (1.18) | 5.04 (1.08) | | 0.3 |
| **High-density lipoprotein(HDL)** | 1.42 (0.46) | 1.43 (0.46) | 1.41 (0.46) | | 0.4 |
| **Hemoglobin A1c** | 5.76 (0.84) | 5.76 (0.84) | 5.78 (0.84) | | 0.6 |
| **Albumin** | 5.50 (2.34) | 5.52 (2.32) | 5.45 (2.37) | | 0.6 |
| **Creatine** | 42.27 (3.21) | 42.27 (3.11) | 42.28 (3.45) | | >0.9 |
| **Blood urea nitrogen(BUN)** | 83.53 (38.20) | 83.30 (38.39) | 84.10 (37.75) | | 0.6 |
| **Aspartate aminotransferase (AST)** | 25.61 (11.86) | 25.56 (10.77) | 25.71 (14.20) | | 0.8 |
| **Alanine aminotransferase(ALT)** | 23.91 (15.71) | 23.83 (14.28) | 24.11 (18.78) | | 0.8 |
| **Triglycerides** | 1.84 (1.74) | 1.85 (1.93) | 1.82 (1.16) | | 0.8 |
| ^1^n (unweighted) (%(weighted)); Mean (SD) or Frequency (Percentage) | | | |  |  |
| ^2^Pearson's X^2: Rao & Scott adjustment; Design-based t-test | | | |  |  |

PIR= Poverty-to-Income Ratio; BMI=Body Mass Index;

Table 2- LASSO regression coefficients for variable selection in the training set.

| **Variables** | **Coefficient** |
| --- | --- |
| (Intercept) | -1.55361 |
| Age group20-44 | 0 |
| Age group >=65 | -0.34871 |
| Gender-Female | 0 |
| Race-Other Hispanic | 0 |
| Race-Non-Hispanic White | 0 |
| Race-Non-Hispanic Black | 0 |
| Race-Other - Including Multi-racial | 0 |
| Education-9-11th Grade | 0 |
| Education-High School Grad or equivalent | 0 |
| Education-Some College or AA degree | 0 |
| Education-College Graduate or above | -0.05243 |
| PIR group1.3~3.5 | 0 |
| PIR group >=3.5 | -0.54052 |
| Marriage-Widowed | 0 |
| Marriage-Divorced | 0 |
| Marriage-Separated | 0 |
| Marriage-Never married | 0 |
| Marriage-Living with partner | 0 |
| BMI -Normal Weight | 0 |
| BMI -Overweight | 0 |
| BMI -Obesity | 0 |
| Diabetes-No | 0 |
| Diabetes-Borderline | 0 |
| Coronary heart disease-No | 0 |
| Stroke-No | 0 |
| Asthma-No | 0 |
| Arthritis-No | 0 |
| Congestive heart-failure-No | -0.18514 |
| Angina-No | 0 |
| Heart attack-No | 0 |
| Chronic bronchitis-No | 0 |
| Thyroid problem-No | 0 |
| Sleep duration-7~9h | 0 |
| Sleep duration >9h | 0 |
| Sleep disorder- No | -0.7444 |
| Smoking status-Former | 0 |
| Smoking status-Never | -0.01238 |
| Drinking status-Former | 0 |
| Drinking status-Never | 0 |
| Hypertension-No | 0 |
| Age at cancer diagnosis | -0.00306 |
| Cancer duration | 0 |
| Number of cancer- Two | 0 |
| Number of cancer-Multiple | 0.043741 |
| Type of cancer- Genitourinary | 0 |
| Type of cancer- Breast | 0 |
| Type of cancer- Gynecological | 0.171967 |
| Type of cancer- Digestive/Gastrointestinal | 0 |
| Type of cancer- Skin (don't know what kind) | 0 |
| Type of cancer- Melanoma | 0 |
| Type of cancer- Other | 0 |
| Total cholesterol | 0 |
| High-density lipoprotein (HDL) | 0 |
| Hemoglobin A1c | 0.083659 |
| Blood urea nitrogen (BUN) | 0 |
| Albumin | 0 |
| Creatine | 0 |
| Aspartate aminotransferase (AST) | 0.001115 |
| Alanine aminotransferase (ALT) | 0 |
| Triglycerides | 0 |

LASSO= Least Absolute Shrinkage and Selection Operator;

PIR= Poverty-to-Income Ratio; BMI=Body Mass Index.

Table S3- Absolute risk differences (ARD) for depression associated with predictors identified

| **Variables** | **ARD** | **95% CI** | **P-value** |
| --- | --- | --- | --- |
| **Age** |  |  |  |
| 45-64 | — | — | — |
| 20-44 | -1.34% | -6.77%, 4.21% | 0.638 |
| >=65 | -7.97% | -11.83%, -4.34% | **<0.001** |
| **Education** |  |  |  |
| Less Than 9th Grade | — | — | — |
| 9-11th Grade | -7.70% | -14.96%, -0.54% | **0.032** |
| High School Graduate or equivalent | -7.60% | -14.22%, -1.16% | **0.027** |
| Some College or AA degree | -6.50% | -13.40%, 0.46% | 0.064 |
| College Graduate or above | -9.61% | -17.20%, -2.43% | **0.011** |
| **PIR** |  |  |  |
| <1.3 | — | — | — |
| 1.3~3.5 | -5.75% | -10.27%, -1.32% | **0.010** |
| >=3.5 | -10.81% | -15.20%, -6.43% | **<0.001** |
| **Congestive heart failure** |  |  |  |
| Yes | — | — | — |
| No | -10.80% | -18.91%, -3.37% | **0.008** |
| **Sleep disorder** |  |  |  |
| Yes | — | — | — |
| No | -9.47% | -12.72%, -6.21% | **<0.001** |
| **Smoking status** |  |  |  |
| Current | — | — | — |
| Former | -7.11% | -12.15%, -2.52% | **0.005** |
| Never | -8.05% | -13.02%, -3.24% | **0.001** |
| **Number of cancer** |  |  |  |
| One | — | — | — |
| Two | 5.15% | -0.40%, 11.23% | 0.086 |
| Multiple | 28.21% | 4.55%, 49.75% | **0.018** |

ARD= Absolute risk difference; PIR= Poverty-to-Income Ratio

Table S4- Multivariable logistic regression results after multiple imputation for variables retained by LASSO

| **Variables** | **OR** | **95% CI** | **P-value** |
| --- | --- | --- | --- |
| **Age** |  |  |  |
| 45-64 | — | — |  |
| 20-44 | 1.04 | 0.67, 1.62 | 0.864 |
| ≥65 | 0.37 | 0.24, 0.56 | **<0.001** |
| **Education** |  |  |  |
| Less Than 9th Grade | — | — |  |
| 9-11th Grade | 0.70 | 0.40, 1.22 | 0.213 |
| High School Graduate or equivalent | 0.60 | 0.34, 1.06 | 0.077 |
| Some College or AA degree | 0.56 | 0.32, 0.98 | **0.044** |
| College Graduate or above | 0.35 | 0.18, 0.70 | **0.003** |
| **PIR** |  |  | **<0.001** |
| <1.3 | — | — |  |
| 1.3~3.5 | 0.56 | 0.38, 0.81 | **0.002** |
| >=3.5 | 0.30 | 0.19, 0.49 | **<**0.001 |
| **Congestive heart failure** |  |  |  |
| Yes | — | — |  |
| No | 0.54 | 0.32, 0.91 | **0.02** |
| **Sleep disorder** |  |  |  |
| Yes | — | — |  |
| No | 0.27 | 0.19, 0.39 | **<0.001** |
| **Smoking status** |  |  |  |
| Current | — | — |  |
| Former | 0.48 | 0.30, 0.77 | **0.002** |
| Never | 0.47 | 0.30, 0.72 | **0.001** |
| **Number of cancer** |  |  |  |
| One | — | — |  |
| Two | 1.30 | 0.75, 2.26 | 0.355 |
| Multiple | 4.45 | 1.30, 15.21 | **0.017** |
| **Cancer type** |  |  |  |
| Digestive/Gastrointestinal | — | — | - |
| Skin (non-melanoma) | 0.86 | 0.45, 1.64 | 0.657 |
| Genitourinary | 0.89 | 0.55, 1.46 | 0.656 |
| Breast | 1.09 | 0.64, 1.86 | 0.745 |
| Gynecological | 1.11 | 0.64, 1.93 | 0.712 |
| Skin (unknown kind) | 0.94 | 0.47, 1.87 | 0.859 |
| Melanoma | 1.19 | 0.51, 2.78 | 0.691 |
| Other | 0.65 | 0.39, 1.10 | 0.108 |
| **Hemoglobin A1c** | 1.14 | 0.99, 1.32 | 0.059 |
| **Angina** |  |  |  |
| Yes | — | — |  |
| No | 0.74 | 0.39, 1.38 | 0.341 |
| **Arthritis** |  |  |  |
| Yes | — | — |  |
| No | 0.78 | 0.55, 1.11 | 0.168 |
| **Asthma** |  |  |  |
| Yes | — | — |  |
| No | 0.91 | 0.59, 1.41 | 0.680 |
| **Chronic bronchitis** |  |  |  |
| Yes | — | — |  |
| No | 0.97 | 0.58, 1.63 | 0.922 |
| **Coronary heart disease** |  |  |  |
| Yes | — | — |  |
| No | 0.62 | 0.36, 1.06 | 0.08 |
| **Stroke** |  |  |  |
| Yes | — | — |  |
| No | 0.66 | 0.42, 1.05 | 0.082 |

PIR= Poverty-to-Income Ratio; LASSO= Least Absolute Shrinkage and Selection Operator.
